# Novel Antioxidant Self-Assembled Peptides Extracted from *Azumapecten farreri* Meat: *In Vitro*- and *In Silico*-Assisted Identification

**DOI:** 10.3390/antiox13070790

**Published:** 2024-06-28

**Authors:** Shuang Zheng, Ronghua Cui, Dingyi Yu, Yanxiang Niu, Xuehan Wu, Faming Yang, Jingdi Chen

**Affiliations:** 1 Marine College, Shandong University, Weihai 264209, China; 202100810142@mail.sdu.edu.cn (S.Z.); 201900810068@mail.sdu.edu.cn (R.C.); 202000810156@mail.sdu.edu.cn (D.Y.); 202100810117@mail.sdu.edu.cn (Y.N.); wuxuehan120500@163.com (X.W.); 2 Shandong Laboratory of Advanced Materials and Green Manufacturing, Yantai 265599, China

**Keywords:** *Azumapecten farreri*, self-assembled peptides, antioxidant peptides, molecular docking

## Abstract

Previous studies have found that the self-assembled supramolecules of *Azumapecten farreri* meat peptides have antioxidant effects. Therefore, this study aims to isolate and identify novel antioxidant peptides with self-assembly characteristics and analyze their structure–activity relationship through molecular docking and molecular dynamics simulation. The in vitro results show that as the purification steps increased, the antioxidant activity of peptides became stronger. Additionally, the purification step did not affect its pH-responsive self-assembly. Using LC-MS/MS, 298 peptide sequences were identified from the purified fraction PF1, and 12 safe and antioxidant-active peptides were acquired through in silico screening. The molecular docking results show that they had good binding interactions with key antioxidant-related protein ligands (KEAP1 (Kelch-like ECH-associated protein 1) and MPO (myeloperoxidase)). The peptide QPPALNDSYLYGPQ, with the lowest docking energy, was selected for a 100 ns molecular dynamics simulation. The results show that the peptide QPPALNDSYLYGPQ exhibited excellent stability when docked with KEAP1 and MPO, thus exerting antioxidant effects by regulating the KEAP1-NRF2 pathway and inhibiting MPO activity. This study further validates the antioxidant and self-assembling properties of the self-assembled supramolecules of *Azumapecten farreri* meat peptide and shows its potential for developing new, effective, and stable antioxidants.

## 1. Introduction

Humans are constantly troubled by various oxidation issues, such as skin aging and lipid oxidation in meat products. These problems can affect quality of life and even threaten human health [1]. According to the theory of free radicals, when the accumulation of reactive oxygen species (ROS) in the body’s cells exceeds the tolerance of the cellular antioxidant defense system, it disrupts the balance between oxidants and antioxidants, leading to oxidative damage, aging, and an indirect relationship with diseases [2]. In the past thirty years, research on antioxidants has gained significant momentum [3]. Therefore, developing an economical and highly compliant new type of antioxidant has become a research hotspot.

Scallops, which have high economic value due to their rich protein and other nutritional substances, have become highly popular bivalves globally [4]. Among them, the *Azumapeten farreri* (i.e., *Chlamys farreri*), mainly distributed in China and the coastal areas of Northeast Asia, is considered a significant marine resource due to its fast growth, delicious meat, and abundant nutrients [5]. However, the processing and utilization of *Azumapecten farreri* still mainly focuses on primary processed products such as canned and dried goods. For example, although the mantle of scallops is edible and rich in protein, it is often overlooked as a low-value byproduct [6]. That hinders the effective utilization of scallop meat, resulting in its low value-added degree.

It is well known that compared to terrestrial organisms, the extreme marine environment (pressure, light, temperature, and salinity) has led to the structural novelty and functional specificity of marine bioactive substances [7]. Among others, the research of marine proteins/peptides with excellent physicochemical and functional properties has garnered much attention [8]. In addition, antioxidant peptides prepared from marine organisms or by-products using the most common and relatively safe reaction conditions and mild and controlled enzymatic methods are highly regarded for their naturalness, safety, and nutritional properties [9,10,11,12,13]. Therefore, obtaining functional substances from the high-quality protein in *Azumapecten farreri* meat through enzymatic hydrolysis is undoubtedly an effective strategy to enhance its added value. For instance, the antioxidant enzymatic hydrolysates from the mantle of *Azumapecten farreri* and the self-assembling peptide hydrogels with metal ions and temperature responsiveness have been reported [14,15,16]. Furthermore, we had successfully prepared pH-responsive self-assembled peptide supramolecules from *Azumapecten farreri* meat with antioxidant, procoagulant, and wound-healing properties that can be orally taken (abbreviated as AAPs, i.e., active polypeptides from *Azumapecten farreri* meat) [17]. Importantly, self-assembled peptides can effectively enhance the stability of peptides, therefore there is no need to explore conservation strategies for its activity [9]. However, the characteristics of its antioxidant peptide sequence are still unclear.

Most importantly, the most critical antioxidant self-assembling peptides were rapidly and precisely screened and identified from numerous peptide sequences [12]. Therefore, we aimed to purify the antioxidant components of AAPs with column chromatography and in vitro antioxidant experiments and verify their pH-responsive self-assembly properties based on SEM; then, identify and screen the peptides with antioxidant effects from them using LC-MS/MS and in silico technology. Finally, the molecular mechanisms of the antioxidant peptides associated with myeloperoxidase MPO and the KEAP1-NRF2 pathway were explored by molecular docking and molecular dynamics simulation techniques. This study provides new insights into the development and high-value utilization of *Azumapecten farreri* meat. It provides a structural and theoretical basis for identifying and investigating the molecular mechanisms of antioxidant peptides in AAPs.

## 2. Materials and Methods

### 2.1. Materials

*Azumapecten farreri* meat was purchased from the Diwanggong Aquatic Market, Huancui District, Weihai City, Shandong Province, China, and animal protease (3 × 10^5^ U/g) was obtained from Pangbo Bioengineering Co., Ltd. (Nanning, China). The 1,1-Diphenyl-2-pyridinylhydrazine (DPPH) and 2,2′-azido-bis (3-ethylbenzothiazoline-6-sulfonic acid) diammonium salts (ABTS) were purchased from Beyotime Biotechnology Co., Haimen, China. All other reagents were of analytical grade.

### 2.2. Preparation and Purification of AAPs

AAPs have been prepared according to our previously described method [17]. Briefly, *Azumapecten farreri* meat was subjected to a controlled enzymatic process (hydrolysis reaction with animal protease at 50 °C, pH 7.0, with an enzyme addition of 1000 U/g (shellfish meat) for 3–5 h) to prepare the enzymatic products. Next, the enzyme was inactivated, and centrifuged, and the supernatant was passed through an ultrafiltration system (GCM-S-02L, GUOCHU Technology, Xiamen, China) and an ultrafiltration membrane (GC-UF0031 made of PES, GE, Boston, MA, USA) to collect the <3 KDa fraction, concentrated, and lyophilized.

Then, an AKTA protein purification system (AKTA Explorer10, GE, Boston, MA, USA, USA) was operated under the following conditions [18]: upper-pressure limit of 1.0 MPa, flow rate of 1.0 mL/min, UV detection wavelength of 280 nm, and a mobile phase of ultrapure water for pump A. The AAPs were dissolved in the ultrapure water and then filtered through a 0.22 μm membrane using a Sephadex G-25 column (GE, Boston, Boston, MA, USA) to obtain 4 fractions (named F1, F2, F3, and F4), which were lyophilized and stored.

Finally, the best antioxidant fractions of the above four fractions were dissolved in ultrapure water, then filtered through 0.22 μm membrane and separated and purified using an ion exchange chromatographic column (Label No. 14001501-EG, HiTrap^TM^ Capto^TM^ MMC, Cytiva, Washington, DC, USA) at a flow rate of 0.2 mL/min. The UV detection wavelength was 220 nm, and one purified fraction was obtained and named as PF1, which was lyophilized and stored.

### 2.3. In Vitro Antioxidant Activity Assay

The DPPH (1,1-diphenyl-2-trinitrophenylhydrazine) and ABTS^+^ (Diammonium 2,2′-azino-bis(3-ethylbenzothiazoline-6-sulfonate)) scavenging methods refer to Supplementary Materials and Methods [7].

### 2.4. LC-MS/MS Analysis

Detection and analysis experiments were entrusted to Sangon Biotech (Shanghai) Co. (Shanghai, China). Briefly, peptide PF1 samples were desalted on a nanoViper C18 pre-column (3 μm, 100 Å, Agilent Technologies, Santa Clara, CA, USA), dried by centrifugation, and re-dissolved in 100 μL of Nano-LC (Ultimate 3000 system, Thermo Fisher Scientific, New York, NY, USA) in mobile phase A (0.1% formic acid/water). The mass spectrometry was performed on a ThermoFisher Q Exactive system (ThermoFisher, Waltham, MA, USA) coupled with a nanolitre-spray Nano Flex ion source (ThermoFisher, Waltham, MA, USA) with a spray voltage of 1.9 kV. The transfer tube was heated at 275 °C.

### 2.5. Characterization of pH-Responsive Self-Assembly Morphology

The pH-responsive self-assembly morphology of peptide PF1 samples was characterized according to the previously described method [17]. In brief, solutions of peptide PF1 samples at a concentration of 1 mg/mL, with pH values of 4.0, 7.0, and 8.0, were prepared separately. They were then vortexed, self-assembled for 30 min, and spotted on clean, freshly cleaved silicon wafers. Finally, the samples were gold-suspended and observed in the SEM (Nova NanoSEM 450, FEI, Hillsboro, OR, USA) sample observation room.

### 2.6. In Silico-Assisted Screening of Novel Antioxidant Peptides and Physicochemical Analysis

In this study, we predicted the bioactivity of peptides using the Peptide Ranker server (http://distilldeep.ucd.ie/PeptideRanker/, accessed on 10 April 2024), and after screening peptides with scores > 0.5, combined with structure–activity relationship analysis, we predicted the bioactivity of peptides using Toxin Pred (https://webs.iiitd.edu.in/raghava/toxinpred/design.php, accessed on 12 April 2024), AnOxPePred—1.0 (https://services.healthtech.dtu.dk/services/AnOxPePred-1.0/, accessed on 13 April 2024), to predict bioactive peptide toxicity and antioxidant activity. We used AllerTOP (https://www.ddg-pharmfac.net/AllerTOP/, accessed on 14 April 2024) to predict the potential sensitization of the peptides and PASTA 2.0 (http://protein.bio.unipd.it/pasta2/, accessed on 15 April 2024) to analyze the molecular stability of the peptides and screen for peptides with non-toxicity, non-sensitization, no self/co-aggregation tendency, and promising peptides to be developed as novel antioxidants. Furthermore, admetSAR (http://lmmd.ecust.edu.cn/admetsar2, accessed on 15 April 2024) was used to predict the absorption, distribution, metabolism, excretion, and toxicity (ADMET) properties of the peptides.

### 2.7. Molecular Docking

The semi-flexible molecular docking between peptides derived from *Azumapecten farreri* meat and KEAP1, MPO was conducted separately using AutoDock Vina (http://vina.scripps.edu/, accessed on 5 May 2024). Prior to the docking procedure, crystal structures of KEAP1 (PDB ID: 2FLU) and MPO (PDB ID: 3F9P) were retrieved from RCSB PDB (http://www.rcsb.org/, accessed on 5 May 2024). The structure of the peptide was drawn using ChemDraw 3D software (CambridgeSoft Co., Ltd., USA, accessed on 6 May 2024). Molecular docking-ready peptides (ligands) were prepared using AutoDock Vina software (accessed on 7 May 2024). Preprocessing of the crystal structures of target proteins was required, including removing water molecules and adding hydrogen atoms and charges. Biovia Discovery Studio and PyMol tools (Pymol 2.5.5, Schrödinger, Inc., New York, NY, USA, accessed on 8 May 2024) were utilized for visualizing these interactions to obtain more comprehensive information about the interactions between proteins and peptides [19,20,21].

### 2.8. Molecular Dynamics Simulation

Based on the results of molecular docking, QPPALNDSYLYGPQ-KEAP1 and QPPALNDSYLYGPQ-MPO were selected to perform molecular dynamics simulations to explore their conformational changes, respectively. In this study, the AMBER ff99SB-ILDN force field of GROMACS 2023 was used to simulate the system for 100 ns. During the simulation, all bonds were constrained using the LINCS algorithm with an integration step of 2 fs. Electrostatic interactions were calculated using the particle mesh Ewald (PME) method with the non-bonding interaction cut-off set to 10 Å. The simulation temperature was controlled by the V-rescale temperature coupling method to 300 K and the Parrinello-Rahman method to control the pressure to 1 Å. The simulation was performed using the V-rescale temperature coupling method. The time step was 2 fs, and snapshots of the conformation were taken every 1.0 ps. At the end of the simulation, GROMACS 2023 was used to analyze the trajectories, kinematic features, and energy distribution of the complexes, and the visualization of the simulation results can be done by VMD (VMD1.9.4, accessed on 9 May 2024), Pymol (Pymol 2.5.5, Schrödinger, Inc., New York, NY, USA, accessed on 9 May 2024), and other software.

### 2.9. Data Statistics and Analysis

The experimental data were processed using Origin 2018 and SPSS 20.0 and expressed in the form of mean ± standard deviation (n = 3). One-way analysis of variance (ANOVA) and Duncan’s method were used to analyze the significance of data between groups.

## 3. Results and Discussion

### 3.1. Purification and Antioxidant Activity of Azumapecten farreri Meat Peptides

**Figure 1:**
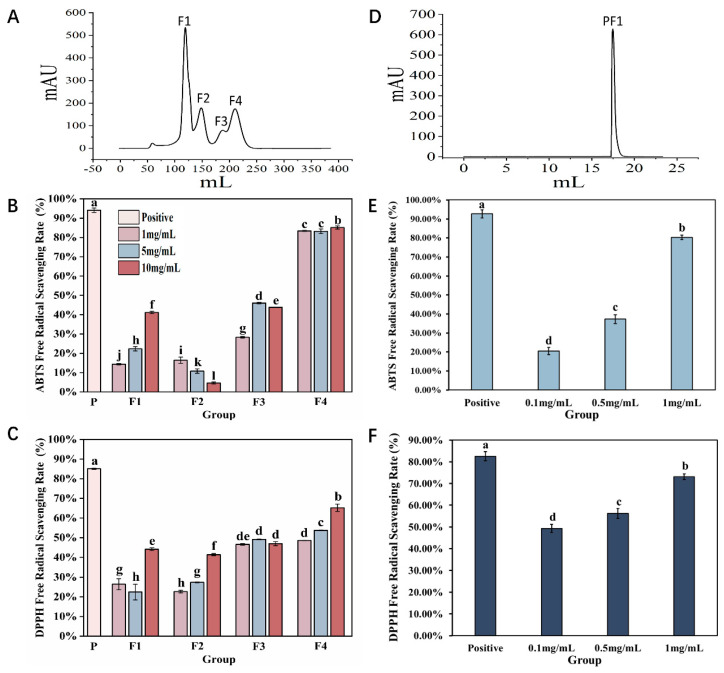
Purification and antioxidant results of *Azumapeten farreri* meat peptide. (**A**) Sephadex G−25 gel column separation and purification spectrum; (**B**,**C**) are the results of ABTS radical scavenging rate and DPPH radical scavenging rate for F1, F2, F3, and F4, respectively; (**D**) Separation and purification of F4 spectrum using ion exchange chromatography column (Label No.14001501-EG, HiTrap^TM^ Capto^TM^ MMC, Cytova, Washington, DC, USA); (**E**,**F**) represent the ABTS radical scavenging rate and DPPH radical scavenging rate of PF1, respectively. The positive control is a 5% ascorbic acid solution. The same superscript letters indicate no significant differences between groups (*p* > 0.05), while different superscript letters indicate significant differences between groups (*p* < 0.05).

Previous studies have shown that AAPs exhibit reducing capabilities and free radical scavenging abilities that are concentration-dependent [17]. To further understand their antioxidant activity, two-step column chromatography methods were used for separation and purification, and their antioxidant activity was evaluated in vitro. Separation results on a Sephadex G-25 gel column are shown in Figure 1A, and a total of four fractions were obtained, which were named F1, F2, F3, and F4. In vitro, antioxidant results indicate that F1, F2, and F3 all exhibit DPPH radical scavenging abilities, but the overall scavenging rate was relatively low. At the same concentration, ABTS radical scavenging was better than DPPH, with F3 showing comparable scavenging effects for both radicals. However, the antioxidant effect of F4 was significantly better than that of the other groups (*p* < 0.05); at a concentration of 10 mg/mL, its scavenging abilities for ABTS and DPPH radicals were 85.13 ± 0.01% and 65.21 ± 0.02%, respectively (Figure 1B,C).

Therefore, an ion exchange chromatography column was selected for further purification of the F4 fraction. As shown in Figure 1D, only one fraction was obtained, and named PF1. The results of in vitro antioxidant tests showed that with increasing concentration, the ABTS and DPPH radical scavenging abilities of PF1 continuously improved. Among them, the antioxidant effect was the best at a concentration of 1 mg/mL (*p* < 0.05), comparable to that of F4. As the two-step purification process progressed, the antioxidant effect of the collected fractions improved, indicating that our separation and purification strategy was appropriate and laying a solid foundation and direction for future research.

### 3.2. Identification, Resolution, and Characterization of Antioxidant Self-Assembling Peptides in Azumapeten farreri Meat

**Figure 2:**
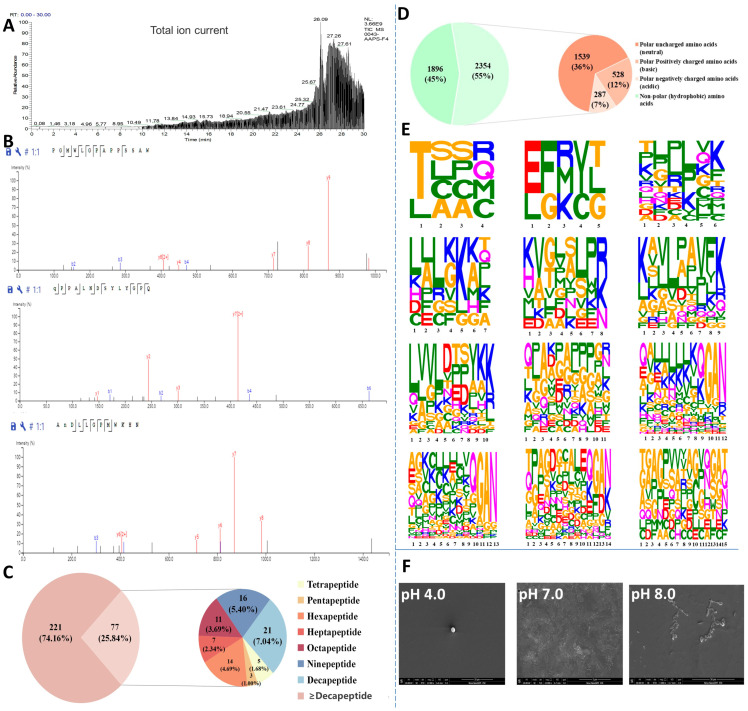
Identification, resolution, and characterization of antioxidant self-assembling peptides (PF1 fraction) from *Azumapeten farreri* meat. (**A**) Total ion spectra of PF1; (**B**) Representative secondary mass spectra of PF1; (**C**) Pie chart of the percentage of peptides of different lengths in PF1 (221 refers to the number of peptide sequences ≥ decapeptide in PF1 and 77 to the number of peptide sequences < decapeptide in PF1.); (**D**) Amino acid distribution of peptide sequences of different lengths in PF1 (It was a pie chart that statistically categorizes the 298 peptide sequences identified from PF1 fractions by LC-MS/MS.) (1896 refers to the statistical quantity of non-polar amino acids (hydrophobic amino acids) in the PF1 peptide sequence, while 2354 refers to the statistical quantity of polar amino acids.); (**E**) Amino acid distribution of peptide sequences of different lengths in PF1; and (**F**) SEM images of in PF1 (self-assembled peptides) with varying pH-responses.

To identify antioxidant peptides from *Azumapecten farreri* meat, the total ion chromatogram of the PF1 fraction (Figure 2A) and the secondary mass spectrum (Figure 2B, representative images) were analyzed by LC-MS/MS. A total of 298 peptides (molecular weight ranging from 428 to 2918 Da, peptide sequence length distribution shown in Figure 2C) were identified from PF1 and ranked according to their area, as shown in Table 1. ALEKFDK, KKKKLKKKSAPLR, GAN, YKK, LPAVFK, KKKKKGAN, and QGAN were the seven characteristic peptides of the C-terminal repeat (Table 1, 32 peptides). In addition, Thr, Glu, Gln, Ala, Lys, and Leu were commonly found at the N-terminus, and Val, Pro, and Asp were commonly seen in the middle of the peptide sequences. Studies have shown that low molecular weight peptides are more likely to have high antioxidant activity and favor intestinal absorption [9,10,22].

Amino acid composition is also a key factor influencing the antioxidant activity of peptides. The amino acid composition of PF1 is shown in Figure 2D, with hydrophobic amino acids (45%), neutral amino acids (36%), alkaline amino acids (12%), and acidic amino acids (7%), in that order. Studies have shown that peptides with high hydrophobic amino acid content can enter target organs smoothly through hydrophobic interactions with cell membranes, and their abundant electrons can be used to quench free radicals. The alkaline amino acids can prevent free radical-induced oxidation of unsaturated fatty acids [23]. As shown in Figure 2E, the amino acid distribution of peptides with peptide lengths of 15 peptides and below in PF1 is demonstrated, and a more significant amino acid character indicates a higher proportion of that amino acid. The figure shows that Thr, Lys, Leu, Gly, Ala, Pro, Glu, and Gln account for a large proportion of the amino acid composition of the peptides in PF1. The presence of these amino acids may contribute to its antioxidant capacity [24]. Most importantly, the SEM results show that PF1 still possesses pH-responsive self-assembly properties (Figure 2F), consistent with previous studies [17].

### 3.3. In Silico Screening and Physicochemical Property Evaluation of Peptide Segments

**Figure 3:**
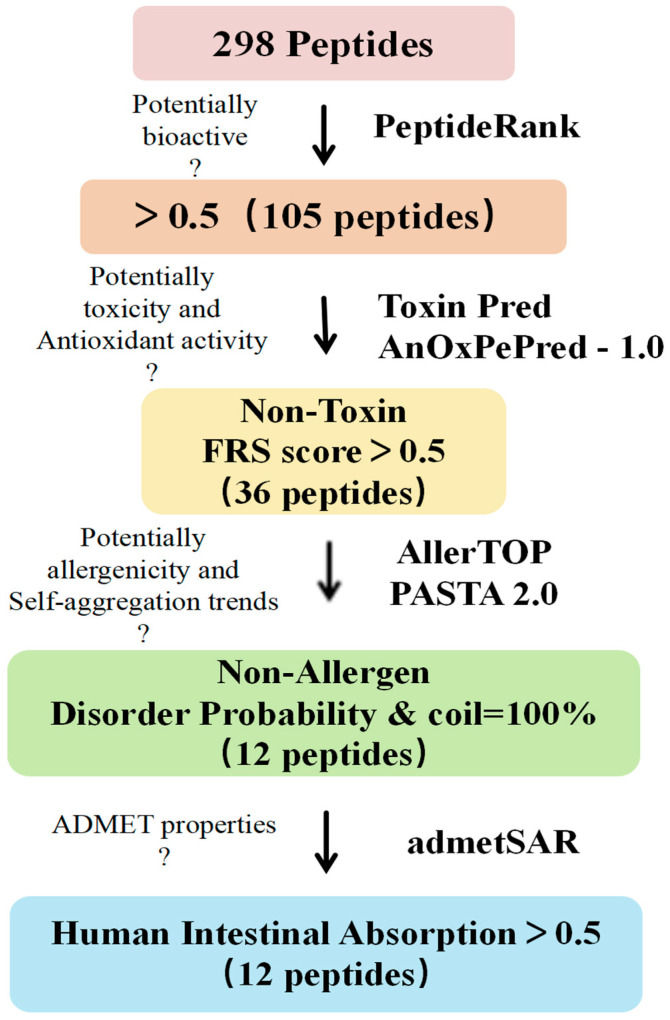
Procedure for in silico screening of peptides and evaluation of physicochemical properties. Bioactive peptides were screened sequentially based on PeptideRank, ToxinPred predicted toxicity, AnOxPePred-1.0 predicted antioxidant activity, AllerTOP predicted allergenicity, PASTA2.0 predicted molecular stability (to determine whether there is a trend towards self-/co-aggregation), and admetSAR predicted ADMET properties to screen for non-toxicity and non-allergenicity, highly disordered, no auto-/co-aggregation tendency and orally deliverable antioxidant peptides.

As shown in Figure 3, we screened 298 peptides for biological activity, antioxidant activity, toxicity, sensitization, stability, and their absorption and metabolic properties using in silico assistance. The results show that among the 298 peptides, 105 peptides with a PeptideRanker score ≥ 0.5 exhibited biological activity [25] (Table S1); 36 peptides exhibited non-toxicity and antioxidant activity [26] (FRS score > 0.5, Table S1), and 12 peptide sequences were screened for non-allergenicity and molecular stability [27] (PASTA 2.0, Table S2).

Finally, the absorption, distribution, metabolism, excretion, and toxicity (ADMET) properties of the above 12 peptide sequences by admetSAR are crucial for their further use, mainly including blood–brain barrier (BBB), human intestinal absorption (HIA), Caco-2 penetration, metabolism properties [28], toxicity, and potential skin sensitization by topical administration (Table 2 and Table S3). admetSAR predictions indicate that the 12 peptides have high Caco-2 permeability and no interaction with CYP450 enzyme, suggesting that the efficacy and safety of these peptides will not be affected by CYP450 enzyme and that they are non-substrate or non-inhibitors of metabolism. Prediction of potential skin sensitization by topical administration indicates that these 12 peptides have a low potential to cause skin sensitization and can be used as active ingredients in skin creams, masks, and other cosmetic products. The HIA results show that the gastrointestinal absorption of these 12 peptides was >50%, and three of them, PGMWLGPAPPSSAW, GAMPASAKAPPGWEP, and ANDLLGPMWKHN, showed excellent gastrointestinal absorption properties and could be used for oral administration.

### 3.4. Molecular Docking Screening of Antioxidant Peptides in Azumapecten farreri Meat

It is widely accepted in the academic community that the KEAP1-NRF2/ARE signaling pathway is currently the most important endogenous antioxidant signaling pathway in the human body. The mechanism is that oxidative stress and electrophilic substances can alter the conformation of KEAP1, leading to the dissociation and activation of NRF2 from KEAP1. Once activated, NRF2 enters the cell nucleus and binds to ARE, and its activation can reduce oxidative damage caused by external stimuli [29,30,31]. Similarly, myeloperoxidase (MPO), a specific marker of myeloid cells, is an enzyme that stimulates oxidative stress in a wide range of inflammatory diseases and is closely associated with the onset and progression of many human diseases [32,33,34,35]. Thus, we performed molecular docking with KEAP1 and MPO as receptor proteins and 12 peptide sequences as ligand molecules, which were used to screen antioxidant-active peptides.

**Figure 4:**
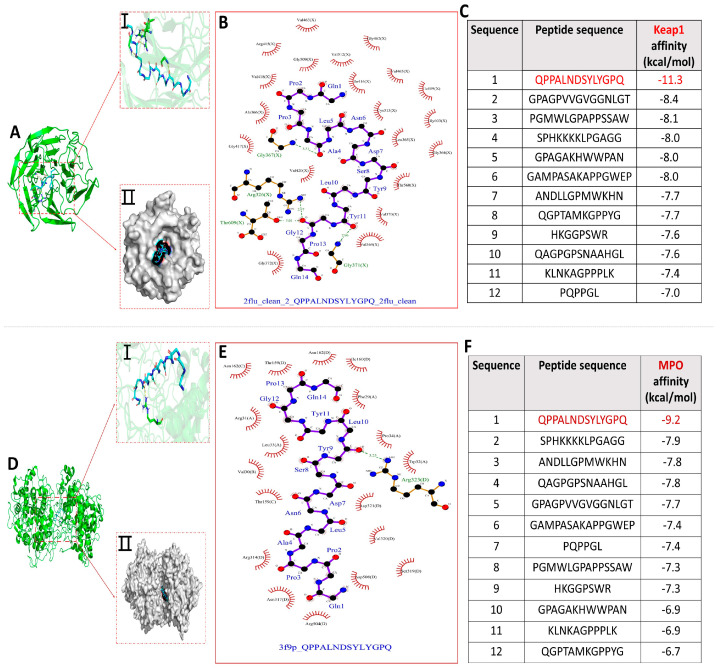
Molecular docking results of 12 peptides with KEAP1 and MPO, respectively. (**A**) Visualization of the interaction between peptide QPPANDSYLYGPQ and KEAP1 active site; (**B**) 2D interaction plot of peptide QPPALNDSYLYGPQ docked with KEAP1; (**C**) Docking energy results of 12 peptides with KEAP1; (**D**) Visualization of the interaction between peptide QPPANDSYLYGPQ and MPO active site; (**E**) Peptide 2D interaction plot of QPPALNDSYLYGPQ docked with MPO; (**F**) Docking energy results of 12 peptides with MPO.

The molecular docking binding energy results of each peptide with KEAP1 and MPO are shown in Figure 4C,F. All binding energies were below −5.0 kcal/mol, the more negative binding affinity signifies more robust binding interactions between the 12 peptides and the receptor (KEAP1 and MPO) [32]. Notably, compared with the other 11 peptides, peptide QPPALNDSYLYGPQ had the lowest docking energies with both KEAP1 and MPO, −11.3 kcal/mol and −9.2 kcal/mol, respectively, and the optimal binding models are shown in Figure 4A,D. Dimethyl fumarate (DMF) has been shown to disrupt the binding interaction between NRF2 and KEAP1, thereby activating the ARE pathway. The binding score of DMF is −3.0 kcal/mol. The binding affinity of 12 peptides to KEAP1 is much lower than it, indicating that peptides can effectively disrupt the binding interaction between NRF2 and KEAP1, thereby activating the ARE pathway, while also inhibiting MPO activity to exert antioxidant effects [32]. The optimal docking active sites of peptide QPPALNDSYLYGPQ with KEAP1 and MPO, respectively, are shown in Figure 4A(I,II),D(I,II).

The interaction of peptide QPPALNDSYLYGPQ-KEAP1 was shown in 2D docking view to form four pairs of hydrogen bonds (Ala4−Gly367, Gly12−Arg326, Gly12−Thr609, and Tyr11−Gly371) and the amino acids of peptide QPPALNDSYLYGPQ (Pro2, Pro3, Leu10, Glu14, Gln1, Asn6, Asp7, Tyr9, Tyr11, Ala4, Ser8, and Gly12) with amino acid residues of KEAP1 (Val418, Ala366, Gly417, Val420, Gly372, Val369, Ile416, Cys513, Leu365, Thr560, Val370, Gly364, Gly603, Ile559, and Val465) underwent hydrophobic interactions that helped to stabilize the binding of QPPALNDSYLYGPQ-KEAP1 (Figure 4B). Similarly, Figure 4E showed that Leu10 of peptide QPPALNDSYLYGPQ forms a hydrogen-bonding interaction with Arg323 of MPO and that amino acids of peptide QPPALNDSYLYGPQ (Pro2, Pro3, Leu10, Gln1, Asn6, Asp7, Tyr9, Tyr11, Ala4, Ser8, Gly12, Pro13, Leu5, and Gln14) with amino acid residues of KEAP1 (Val30, Thr159, Asn162, Arg31, Leu33, Thr159, Arg314, Asn317, Arg504, Asp508, Ser319, Val320, Asp321, Trp32, Pro34, Phe29, and Ile160) hydrophobic interactions took place between them. Therefore, hydrogen bonding and numerous hydrophobic interactions are key factors in the formation of stable binding of peptide QPPALNDSYLYGPQ to KEAP1 and MPO. Critically, a comparison of the interactions of the peptide QPPALNDSYLYGPQ amino acids with KEAP1 and MPO residues showed that their hydrophobic interactions jointly benefit from the interaction of the peptide QPPALNDSYLYGPQ amino acids (Pro2, Pro3, Leu10, Glu14, Gln1, Asn6, Asp7, Tyr9, and Tyr11) with the receptor.

Using BLAST software (National Center for Biotechnology Information, NCBI, https://blast.ncbi.nlm.nih.gov/Blast.cgi, accessed on 10 June 2024), the 12 obtained peptides were subjected to similarity protein matching searches [36]. Table S4 shows the sequence numbers, names, and sources of 37 proteins with a sequence identity (Per. Ident) of ≥65% compared to the 12 peptides. Among these, 21 proteins have a Per. Ident of ≥90%. Importantly, the sequences of the 12 peptides were highly matched with 26 proteins that have antioxidant functions, including zinc finger protein 469 (XP_034528409.1), catalase/peroxidase HPI (WP_153758351.1), and CmcJ/NvfI family oxidoreductase (MCY4450844.1). The antioxidant activity of the peptide QPPALNDSYLYGPQ, which had the best molecular docking results, is attributed to its high sequence matching with FAD dependent oxidoreductase (KAI1610276.1), FAD dependent oxidoreductase (XP_062674824.1), and CmcJ/NvfI family oxidoreductase (MCY4450844.1). In addition, this also contributes to the validity of the above findings as well as to the elucidation of the antioxidant mechanism of the 12 peptides.

### 3.5. Molecular Dynamics Modeling of Antioxidant Mechanisms

**Figure 5:**
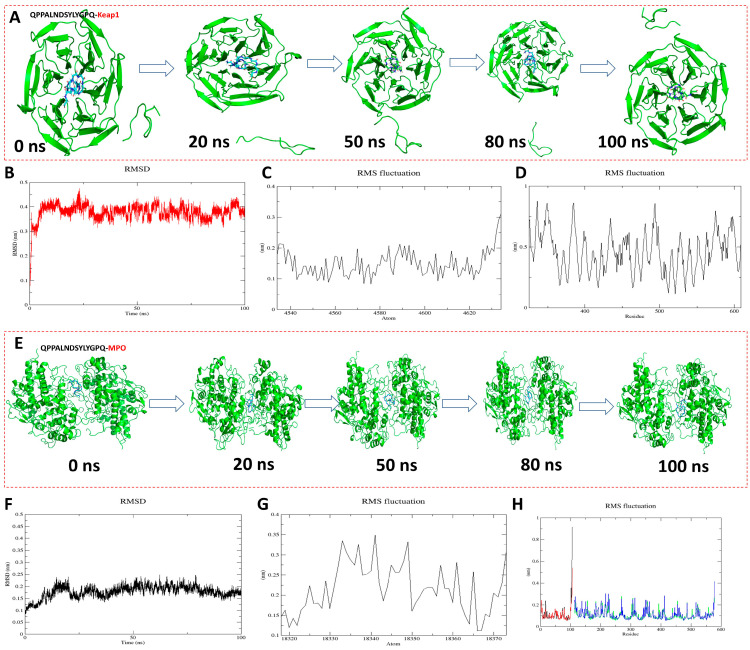
Molecular dynamics simulation results of peptide QPPALNDSYLYGPQ binding to KEAP1 and MPO, respectively. (**A**,**E**) Conformations of different simulated nodal systems; (**B**,**F**) Root mean square deviation (RMSD); (**C**,**G**) Root mean square fluctuation (RMSF) results of protein backbone X; (**D**,**H**) Root mean square fluctuation (RMSF) results of peptide QPPALNDSYLYGPQ.

To further analyze the antioxidant mechanism and stability of QPPALNDSYLYGPQ-KEAP1 and QPPALNDSYLYGPQ-MPO, molecular dynamics simulations were performed up to 100 ns (Figure 5). As can be seen from the conformational pictures, the protein backbone X and the peptide ligand remained in a relatively stable state as the simulation proceeded (Figure 5A,E). The figurative results of the structural changes of the complexes during the simulation can be obtained by analyzing the RMSD and RMSF [37]. The RMSD fluctuations of QPPALNDSYLYGPQ-KEAP1 and QPPALNDSYLYGPQ-MPO were respectively in the range of 0.3–0.45 nm (Figure 5B) and 0.1–0.25 nm, and these results indicate that they both reached stable equilibrium within 100 ns of the simulation. The RMSF was evaluated by assessing the RMSF within 100 ns to reveal local changes in the protein structure. The results show that peptide QPPALNDSYLYGPQ has the same RMSF trend as KEAP1 and MPO during the simulation, indicating that the entire protein structure is stable and the peptide molecule does not change much upon binding to the protein [38]. Thus, peptide QPPALNDSYLYGPQ is able to stably bind to the active pockets of KEAP1 and MPO and is the best antioxidant peptide among AAPs [39].

## 4. Conclusions

In the present study, we purified the self-assembled supramolecules of *Azumapecten farreri* meat peptides and evaluated their in vitro antioxidant activity. Twelve novel peptides, such as QPPALNDSYLYGPQ, GPAGPVVGVGGGNLGT, PGMWLGPAPPSSAW, SPHKKKKKLPGAGG, GPAGAKHWWPAN, and GAMPASAKAPPGWEP, were screened by LC-MS/MS and machine-assisted screening of the optimal fractions. The conformational relationships and antioxidant mechanisms of the 12 peptides, with typical target proteins KEAP1 and MPO, were further investigated with the help of molecular docking and molecular dynamics simulation techniques, respectively. The results show that the peptides may exert antioxidant effects through stable binding to the active sites of the target proteins via hydrogen bonding and hydrophobic interactions, among which only the peptide QPPALNDSYLYGPQ was more readily bound to the two target proteins in 100 ns. In the future, we will further synthesize the peptide to study its antioxidant mechanism.

## Figures and Tables

**Table 1:** Main peptide sequences analysis of PF1.

Sequence	Peptide Sequence of PF1	Molecular Mass (Da)	Area
1	TPFVDDF**ALEKFDK**	1634.77	4,737,508
2	TPQNDTFALEKFDK	1634.78	4,737,508
3	FPVDDFTALEKFDK	1634.77	4,737,508
4	TPFNNFTALEKFDK	1634.79	4,737,508
5	ASL**KKKKLKKKSAPLR**	1823.22	3,903,436.8
6	TVAKKKKLKKKSAPLR	1823.22	3,903,436.8
7	TVKKKKKLKKLSAPLR	1823.22	3,903,436.8
8	**LVV**LDTD**YKK**	1192.67	3,880,284.2
9	LVVLDESYKK	1192.67	3,880,284.2
10	LVVLDAKYSSV	1192.67	3,388,490.5
11	VLVLDTDYKK	1192.67	3,388,490.5
12	LVVLCMPYKK	1192.67	3,388,490.5
13	LVVLDTDYKK	1192.66	3,388,490.5
14	GVV**LPAVFK**	928.57	2,675,474
15	LAALPAVFK	928.57	2,675,474
16	AALLPAVFK	928.57	2,675,474
17	LAALVAPFK	928.57	2,675,474
18	KKLF**KKKKKGAN**	1416.93	720,098.4
19	FKKLKKKKKGAN	1416.93	720,098.4
20	KFLKKKKKKGAN	1416.93	720,098.4
21	FKKKLKKKKGAN	1416.93	720,098.4
22	ASKVPLPKPK**GAN**	1305.78	594,084
23	GAKLKLLPP**QGAN**	1305.78	594,084
24	KALHKKLVQGAN	1305.79	594,084
25	LSKKKAPVPGAN	1305.78	594,084
26	TVKLLLLHQGAN	1305.78	594,084
27	FKLTAKKKTGAN	1305.78	594,084
28	FQMTPDDDDQGAN	1416.51	578,595.3
29	FGKCCMEMTQGAN	1416.51	578,595.3
30	QYCCMPEGTQGAN	1416.51	578,595.3
31	QGPGDDSPDCGQGAN	1416.52	578,595.3
32	QYPNSMMCEGGAN	1416.51	578,595.3

**Table 2:** Results of ADMET characterization of peptides.

Sequence	Blood–Brain Barrier	Human Intestinal Absorption	Caco-2 Permeability	CYP Inhibitory Promiscuity	Carcinogens	Acute Oral Toxicity
GPAGAKHWWPAN	0.9886	0.5996	0.9176	0.5920	Non-carcinogens	III
PQPPGL	0.9426	0.6009	0.8636	0.9908	Non-carcinogens	III
PGMWLGPAPPSSAW	0.9897	0.8710	0.8433	0.8507	Non-carcinogens	III
GPAGPVVGVGGNLGT	0.9857	0.5467	0.8242	0.9714	Non-carcinogens	III
QAGPGPSNAAHGL	0.9892	0.6042	0.8501	0.9573	Non-carcinogens	III
GAMPASAKAPPGWEP	0.9602	0.8213	0.8429	0.9473	Non-carcinogens	III
HKGGPSWR	0.8951	0.6568	0.8598	0.9743	Non-carcinogens	III
ANDLLGPMWKHN	0.9938	0.8979	0.8683	0.9229	Non-carcinogens	III
KLNKAGPPPLK	0.9881	0.6617	0.8364	0.9774	Non-carcinogens	III
SPHKKKKLPGAGG	0.9892	0.6042	0.8501	0.9573	Non-carcinogens	III
QPPALNDSYLYGPQ	0.9967	0.7428	0.8551	0.9115	Non-carcinogens	III
QGPTAMKGPPYG	0.9796	0.7835	0.8105	0.9872	Non-carcinogens	III

## Data Availability

Data supporting the reported results are contained within the article or are available from the corresponding author.

## Supplementary Materials

The following supporting information can be downloaded at: https://www.mdpi.com/article/10.3390/antiox13070790/s1, Table S1: Evaluation of toxicity and predicted antioxidant activity of peptides with PeptideRanker score > 0.5; Table S2: Predicted sensitization and molecular stability of peptides; Table S3: Results of ADMET characterization of peptides. Table S4: Results of BLAST searches of peptides.
