# Supplementary material for: Novel Antioxidant Self-Assembled Peptides Extracted from Azumapecten farreri Meat: In Vitro- and In Silico-Assisted Identification
Source: Antioxidants (Basel). 2024 Jun 28;13(7):790. doi: 10.3390/antiox13070790 (PMC11273597; doi:10.3390/antiox13070790)
Supplement: Supplementary file 1 [file antioxidants-13-00790-s001.zip › antioxidants-3038333-supplementary.pdf]

Novel antioxidant self-assembled peptides extracted from the *Azumapecten farreri* meat: *in Vitro* and *in Silico* assisted identification

Shuang Zheng [1], Ronghua Cui [1], Dingyi Yu [1], Yanxiang Niu [1], Xuehan Wu [1], Faming Yang [1,*] and Jingdi Chen [1,2,*]

[1] *Marine College, Shandong University, Weihai 264209, China*

[2] *Shandong Laboratory of Advanced Materials and Green Manufacturing, Yantai 265599, China*

Corresponding Author

*Faming Yang, Ph.D. and Jingdi Chen, Ph.D.

Marine College, Shandong University, No. 180, Wenhuaxi Road, Weihai 264209 China

Tel: +86 6315688303

E-mail addresses: yangfm0123@163.com (F.Y.); jdchen@sdu.edu.cn (J.C.)

Supplementary Materials and Methods

1. Methods

1.1 In vitro antioxidant activity assay

The 1,1-Diphenyl-2-picrylhydrazyl (DPPH) radical scavenging ability was assessed based on previous studies. Briefly, an aliquot of 2 mL of DPPH solution (0.1 mM in 95% methanol) was mixed with 2 mL of samples with varying concentrations. And the ascorbic acid was considered a positive control. Afterwards, the plate was

incubated at room temperature in the dark for 30 min. The absorbance of mixture was monitored at 517 nm using a microplate reader (VersaMax, Molecular Devices, USA).

As described previously, the ABTS radical scavenging activities were assessed. The ABTS+ solution was prepared by mixing ABTS solution (0.7mM) to an equal volume of potassium persulfate (2.45 mM) and incubated at room temperature for 16 h in the dark. Next, the ABTS+ solution was diluted with PBS to the absorbance of 0.70 ± 0.05 at 734 nm. The samples with varying concentrations (5.0 μL) was added to 200 μL of diluted ABTS+ solution and incubated for 10 min in the dark. Following incubation, the absorbance at 734 nm was monitored using a microplate reader (VersaMax, Molecular Devices, USA).

2. Results

Table S1. Evaluation of toxicity and predicted antioxidant activity of peptides with PeptideRanker score > 0.5.

Sequence	Peptideranker	Toxicity	FRS score	CHEL score	pI
CCCCCCCCCCQGAN	0.991967	Non-Toxin	0.40920368	0.21518888	5.67
RGTKSLQVCRGCCMSLLDAQSAPLR	0.980387	Toxin	0.41655898	0.29610553	9.26
ADGALLVNFWAEWCGGPGCK	0.960381	Non-Toxin	0.6081754	0.24146459	4.38
GFDLGF	0.958032	Non-Toxin	0.43508857	0.29055271	3.80
EGGGALLAAFSNAEGWCGGPGCK	0.952824	Non-Toxin	0.57783383	0.24876599	4.54
GVGTVQWLLSMCHNSLLDAQSAPLR	0.952761	Non-Toxin	0.51274353	0.29568455	7.06
AFQCCCYGWAN	0.949206	Toxin	0.54089773	0.17994808	5.79
SCCCCCECMQGAN	0.946907	Toxin	0.38028595	0.23514907	4.00
QPCYCC	0.940084	Toxin	0.53296745	0.29683003	5.79
ASCCCCMCCQGAN	0.939075	Toxin	0.42720819	0.21385992	5.73
QGPGECCMCCQGAN	0.932478	Toxin	0.48949403	0.21173695	4.00
DFGGLGF	0.919063	Non-Toxin	0.48958772	0.29055271	3.80
RVARFACCTTKWSSLLNAAGSALPR	0.90124	Non-Toxin	0.40640137	0.30563051	10.79
GPAGAKHWWPAN	0.899651	Non-Toxin	0.61876446	0.19243154	9.11
TGPGECACCCCQGAN	0.884838	Toxin	0.46769494	0.20921223	4.00
PQPPGL	0.883761	Non-Toxin	0.51725823	0.33184636	5.88
AAHCCCCMMQGAN	0.882333	Toxin	0.50565392	0.29660985	6.98
LFRYL	0.869535	Non-Toxin	0.49765524	0.29248416	9.10
GPLFVGLP	0.869026	Non-Toxin	0.48360866	0.31906638	5.88
GPEPPGGLSGEPGF	0.868208	Non-Toxin	0.56119227	0.33184636	3.80
NCGDGLLGPVCHSGGR	0.867354	Non-Toxin	0.49016756	0.25797865	7.03
ENPADCCCCQGAN	0.856436	Toxin	0.40308061	0.21661852	3.67
LAGPKCYRAF	0.855303	Non-Toxin	0.52044642	0.30438244	9.36
QLGYPGQAPWW	0.85056	Non-Toxin	0.44010532	0.27900964	5.88
PGLLFK	0.841787	Non-Toxin	0.47251096	0.30295384	9.11
GPDTPTGPPWAGPPPASKC	0.831415	Toxin	0.63763618	0.30880904	6.16
LSDSSALSVAKLMCMSLLDAAGSALPR	0.831287	Non-Toxin	0.42231187	0.27691993	6.28
PGAEPVPGSGPQGF	0.831157	Non-Toxin	0.50876051	0.30438244	4.00
ECNGGSVGAPGAAKLFM	0.830338	Non-Toxin	0.48226401	0.30295384	6.32
ADGALLVDFWAEGECGGPGCK	0.824485	Non-Toxin	0.56369764	0.30438244	3.92
ADGALLVDFVSAQWCGGNRN	0.823834	Non-Toxin	0.52353197	0.29639882	4.21
PGMWLGPAPPSSAW	0.807861	Non-Toxin	0.57828629	0.33184636	5.88
SSSAGAFLLMYG	0.803071	Non-Toxin	0.53378248	0.29639882	5.88
SCCCCYMSSQGAN	0.800031	Toxin	0.52044642	0.28276169	5.77
EGCQGAMGPAGF	0.793461	Non-Toxin	0.49871626	0.30438244	4.00
TCCC	0.791454	Non-Toxin	0.43993402	0.26231942	5.79

Sequence	Peptideranker	Toxicity	FRS score	CHEL score	pI
GPAGPVVGVGGNLGT	0.786627	Non-Toxin	0.502563	0.30438244	5.88
LSPSAWTVAGAMVCYSLLDAQSAPL R	0.784328	Non-Toxin	0.54855305	0.26800993	6.15
LSQEGYTVACAAMVLVTLDGAAGTGLPR	0.782544	Non-Toxin	0.46321875	0.25058934	4.38
ECCCCCCSSQGAN	0.776301	Toxin	0.44696745	0.28276169	4.00
SLGGSGFASKGMAASDLSLLNAAGSALPR	0.772879	Non-Toxin	0.3997173	0.30184963	9.10
AAVSCFMCCQGAN	0.756047	Toxin	0.45035028	0.28451738	5.79
NPAGAACMMMQGAN	0.750398	Non-Toxin	0.45125353	0.30752134	5.85
GPACMQRPAGPAGAAGHVKN	0.750201	Non-Toxin	0.49985811	0.26226571	9.55
SAPTAPMRMVYAVCASLLNAQSAPLR	0.748901	Non-Toxin	0.47362524	0.29334745	9.55
GPAGPGPSTASPNKMT	0.748344	Non-Toxin	0.53206414	0.31182522	9.11
GVGTPAALCFWYSGLSLLNAQSALPR	0.747503	Non-Toxin	0.55445963	0.30612811	8.57
LGFAQFVLR	0.743687	Non-Toxin	0.43508857	0.29055271	10.11
CECGGMP	0.736642	Non-Toxin	0.49712491	0.29532823	4.00
MAFASLPR	0.734337	Non-Toxin	0.45273823	0.31906638	10.11
QPGYPNGHLAGPPDSAGR	0.731677	Non-Toxin	0.57383364	0.27563515	7.09
QSLGPLGSKFGT	0.729419	Non-Toxin	0.48360866	0.30848914	9.11
ASLKKKKLKKKSAPLR	0.713918	Non-Toxin	0.44240913	0.30848914	11.48
QAGPGPSNAAHGL	0.713199	Non-Toxin	0.53206414	0.31090766	7.10
ADGALLVNFANAESVCGSSNGP	0.710874	Non-Toxin	0.4195959	0.25250515	3.67
EQPGCCCCDQGAN	0.706805	Toxin	0.47946519	0.30295384	3.67
WTGAVYSLAACAASDLSLLDAQSAPLR	0.704363	Non-Toxin	0.40411007	0.30364242	4.21
ADGALLVSSSWNSWCGGPGCK	0.701196	Non-Toxin	0.55079257	0.23012751	6.13
TGNSCCCCCKDN	0.698772	Toxin	0.44865513	0.28157282	6.06
QPAGSNSPPGL	0.689859	Non-Toxin	0.51725823	0.33184636	5.88
ADAGPSQVGLVNFGF	0.673302	Non-Toxin	0.48360866	0.30438244	3.80
QGPGDDSPDCGQGAN	0.667433	Non-Toxin	0.50544637	0.30898789	3.43
GAMPASAKAPPGWEP	0.66555	Non-Toxin	0.55251133	0.33184636	6.35
HKGGPSWR	0.65451	Non-Toxin	0.50123268	0.30438244	11.01
AEEALLVLLAMGM	0.65354	Non-Toxin	0.43851703	0.29639882	3.80
ANDLLGPMWKHN	0.645446	Non-Toxin	0.54505545	0.31420198	7.09
FGRWEVFQGAN	0.64384	Toxin	0.50481492	0.28494915	6.36
TGASPDGAPGF	0.638093	Non-Toxin	0.49557793	0.30898789	3.80
APQEEADPPFR	0.637214	Non-Toxin	0.49563733	0.33184636	4.14
TVGSPADSAKLGGDFMGSLLDAQSAPLR	0.636317	Non-Toxin	0.44017598	0.2891843	4.43

Sequence	Peptideranker	Toxicity	FRS score	CHEL score	pI
KLNKAGPPPLK	0.633658	Non-Toxin	0.55449826	0.34008321	10.31
SPHKKKKLPGAGG	0.632183	Non-Toxin	0.53724605	0.33023757	10.49
GPAGDCPVAAGTQLVQGV	0.629762	Non-Toxin	0.4341605	0.2674019	3.80
SNNCHDCCQGAN	0.624141	Toxin	0.51274353	0.29923248	5.09
QGNGNLGPYGKP	0.61954	Non-Toxin	0.59610522	0.30438244	8.94
EAGYCLNWQGAN	0.617376	Non-Toxin	0.53682953	0.295874	4.00
QPPALNDSYLYGPQ	0.615384	Non-Toxin	0.61605066	0.33184636	3.80
QQCCCEAPYQGAN	0.612493	Toxin	0.54603744	0.29774734	4.00
AAPGKKKLKAGGAN	0.607734	Non-Toxin	0.47251096	0.30295384	10.49
QTQRGCCGPVGL	0.603098	Toxin	0.50809443	0.30438244	8.39
KVCKVPRGLL	0.599501	Non-Toxin	0.45315677	0.29639882	10.07
KGPPVP	0.596552	Non-Toxin	0.52389091	0.33184636	9.11
KRHVFLLPP	0.591709	Non-Toxin	0.49563733	0.33599371	11.01
GTAPGAPGLPG	0.581543	Non-Toxin	0.51348937	0.31906638	5.88
GPAVENGPAPVAGPYYGVT	0.575441	Toxin	0.65416127	0.2445236	4.00
AALLVAPFK	0.571045	Non-Toxin	0.44725531	0.3043223	9.11
QVRAGAAGAPGAPGCPGY	0.564746	Non-Toxin	0.55312359	0.23313843	8.57
QGPTAMKGPPYG	0.564272	Non-Toxin	0.59100902	0.33184636	8.94
GVGTAPAFRVDHWESLLNAAGSALPR	0.557365	Non-Toxin	0.51730585	0.28409839	7.11
AAGPLGPQTR	0.555994	Non-Toxin	0.50985974	0.31776601	10.11
LLFPKPPAAK	0.554642	Non-Toxin	0.49563733	0.33184636	10.02
GAADAPVPSG	0.549003	Non-Toxin	0.45433319	0.30395761	3.80
YVVLKAWCKRYGHGHALLQK	0.542212	Non-Toxin	0.61118323	0.29586104	9.88
QSADGRAPVGL	0.540746	Non-Toxin	0.44240913	0.29774734	6.19
LVGLLLLLLPGAN	0.540343	Non-Toxin	0.47479019	0.31906638	5.88
AALLPAVFK	0.539334	Non-Toxin	0.45273823	0.31906638	9.11
CQGMLVVRAGLEGCQ	0.539244	Non-Toxin	0.44823074	0.28222927	6.30
QDQAVGLSGPAGGK	0.531077	Non-Toxin	0.5005182	0.30438244	6.19
GPAGDSSREPNGLV	0.520312	Non-Toxin	0.48360866	0.30767852	4.38
YWTQVMLVCAASDLSLLDAQSALPVG	0.513263	Non-Toxin	0.37339091	0.30627972	3.57
VTAGLTKKKKVRKKSLLNASQAPLR	0.512435	Non-Toxin	0.37035742	0.28522667	12.05
QYCCMPEGTQGAN	0.507913	Toxin	0.52615821	0.29532823	4.00
ASKVPLPKPKGAN	0.504684	Non-Toxin	0.47496632	0.31906638	10.31
SAPTWYSLAACAASDLSLLNAQSAPLR	0.504436	Non-Toxin	0.47118801	0.32378328	6.15

Table S2. Predicted sensitisation and molecular stability of peptides.

Sequence	Length	Allergenicity	Disorder Probability (%)	Probability in secondary structure (%)		
				α-helix	β-strand	coil
ADGALLVNFWAEWCGGPGCK	20	Non-Allergen	40	60	0	40
EGGGALLAAFSNAEGWCGGPGCK	23	Non-Allergen	60.86	39.13	0	60.87
GVGTVQWLLSMCHNSLLDAQSAPLR	25	Allergen	100	68	0	32
GPAGAKHWWPAN	12	Non-Allergen	100	0	0	100
PQPPGL	6	Non-Allergen	100	0	0	100
GPEPPGGLSGEPGF	14	Allergen	100	0	0	100
LAGPKCYRAF	10	Allergen	100	0	0	100
PGAEPVPGSGPQGF	14	Allergen	100	0	0	100
ADGALLVDFWAEGECGGPGCK	21	Non-Allergen	52.38	0	28.57	71.43
ADGALLVDFVSAQWCGGNRN	20	Non-Allergen	40	25	20	55
PGMWLGPAPPSSAW	14	Non-Allergen	100	0	0	100
SSSAGAFLLMYG	12	Non-Allergen	100	58.33	0	41.67
GPAGPVVGVGGNLGT	15	Non-Allergen	100	0	20	80
LSPSAWTVAGAMVCYSLLDAQSAPLR	26	Allergen	65.38	65.38	0	34.62
GPAGPGPSTASPNKMT	16	Allergen	100	0	0	100
QPGYPNGHLAGPPDSAGR	18	Allergen	100	0	0	100
QAGPGPSNAAHGL	13	Non-Allergen	100	0	0	100
ADGALLVSSSWNSWCGGPGCK	21	Allergen	52.38	0	19.05	80.95
QPAGSNSPPGL	11	Allergen	100	0	0	100
QGPGDDSPDCGQGAN	15	Allergen	100	0	0	100
GAMPASAKAPPGWEP	15	Non-Allergen	100	0	0	100
HKGGPSWR	8	Non-Allergen	100	0	0	100
ANDLLGPMWKHN	12	Non-Allergen	100	0	0	100
KLNKAGPPPLK	11	Non-Allergen	100	0	0	100
SPHKKKKLPGAGG	13	Non-Allergen	100	0	0	100
QGNGNLGPYGKP	12	Allergen	100	0	0	100
EAGYCLNWQGAN	12	Non-Allergen	100	0	16.67	83.33
QPPALNDSYLYGPQ	14	Non-Allergen	100	0	0	100
KGPPVP	6	Allergen	100	0	0	100
GTAPGAPGLPG	11	Allergen	100	0	0	100
QVRAGAAGAPGAPGCPGY	18	Allergen	100	0	0	100
QGPTAMKGPPYG	12	Non-Allergen	100	0	0	100
GVGTAPAFRVDHWESLLNAAGSALPR	26	Non-Allergen	100	46.15	0	53.85

Sequence	Length	Allergenicity	Disorder Probability (%)	Probability in secondary structure (%)		
				α-helix	β-strand	coil
AAGPLGPQTR	10	Allergen	100	0	0	100
YVVLKAWCKRYGHGHALLQK	20	Non-Allergen	40	65	0	35
QDQAVGLSGPAGGK	14	Allergen	100	0	0	100

Table S3. Results of ADMET characterization of peptides

Sequence	CYP450 2C9 Substrate	CYP450 2D6 Substrate	CYP450 3A4 Substrate	CYP450 1A2 Inhibitor	CYP450 2C9 Inhibitor	CYP450 2D6 Inhibitor	CYP450 2C19 Inhibitor	CYP450 3A4 Inhibitor
GPAGAKHWWPAN	0.7991	0.7322	0.5817	0.8057	0.6475	0.9162	0.7441	0.6410
PQPPGL	0.9040	0.7298	0.5501	0.9507	0.9441	0.9670	0.8372	0.9707
PGMWLGPAPPSSAW	0.7977	0.7613	0.6421	0.8232	0.6708	0.9128	0.6783	0.6427
GPAGPVVGVGGNLGT	0.8965	0.7518	0.5396	0.9152	0.9107	0.9322	0.8528	0.8817
QAGPGPSNAAHGL	0.8550	0.7879	0.5224	0.8968	0.8889	0.9181	0.8406	0.7967
GAMPASAKAPPGWEP	0.8735	0.7803	0.5339	0.8154	0.8174	0.9235	0.8058	0.8806
HKGGPSWR	0.8358	0.7812	0.6571	0.7791	0.9145	0.9273	0.8553	0.8843
ANDLLGPMWKHN	0.8154	0.6977	0.5902	0.8246	0.7316	0.9459	0.7703	0.9502
KLNKAGPPPLK	0.8782	0.7350	0.5269	0.9264	0.9058	0.9429	0.8655	0.9421
SPHKKKKLPGAGG	0.8550	0.7879	0.5224	0.8968	0.8889	0.9181	0.8406	0.7967
QPPALNDSYLYGPQ	0.8226	0.7861	0.5952	0.9569	0.9144	0.8960	0.8578	0.6915
QGPTAMKGPPYG	0.8518	0.7724	0.5259	0.9324	0.9427	0.9173	0.9232	0.9734

Table S4. Results of BLAST searches of peptides

Sequence	Description	Total Score	E value	Per. Ident
GPAGAKHWWPAN	Select seq tpg|HWV25976.1| TPA: catalase/peroxidase HPI [Aeromicrobium sp.]	31.6	41	81.82%
	Select seq ref|WP_153758351.1| catalase/peroxidase HPI [Actinomarinicola tropica]	31.6	41	81.82%
PQPPGL	Select seq ref|WP_160964882.1| LEPR-XLL domain-containing protein [Pseudomaricurvus sp. HS19]	22.3	24037	100.00%
	Select seq dbj|GFS22358.1| myosin-10 [Elysia marginata]	22.3	24039	100.00%
	Select seq ref|XP_034528409.1| zinc finger protein 469 [Ailuropoda melanoleuca]	22.3	24052	100.00%
PGMWLGPAPPSSAW	Select seq tpg|HEV2947438.1| TPA: Gfo/Idh/MocA family oxidoreductase [Gemmataceae bacterium]	30.8	121	100.00%
	Select seq gb|MBY0229886.1| BatA domain-containing protein [Gemmataceae bacterium]	30.8	121	76.92%
	Select seq tpg|HLT06723.1| TPA: Gfo/Idh/MocA family oxidoreductase [Cyclobacteriaceae bacterium]	30.8	121	100.00%
GPAGPVVGVGGNLGT	Select seq gb|KAG5300392.1| C6 transcription factor [Histoplasma ohiense (nom. inval.)]	31.2	102	83.33%
	Select seq ref|WP_215095067.1| zinc-binding dehydrogenase [Streptomyces sp. ISL-1]	29.9	291	76.47%
QAGPGPSNAAHGL	Select seq gb|MCC6518177.1| FAD-dependent oxidoreductase [Tabrizicola sp.]	28.2	819	75.00%
	Select seq gb|MBX7137542.1| 2-oxoglutarate dehydrogenase E1 component [Oligoflexia bacterium]	27.8	1159	90.00%
	Select seq gb|RLM75248.1| myb-related protein Myb4-like [Panicum miliaceum]	27.8	1162	90.00%
GAMPASAKAPPGWEP	Select seq ref|WP_308114163.1| DNA-binding protein [Streptomyces brasiliscabiei]	30.8	145	75.00%
	Select seq gb|MBK5228616.1| serine protease [Actinomycetota bacterium]	30.8	145	71.43%
HKGGPSWR	Select seq tpg|HEY4394499.1| TPA: DCC1-like thiol-disulfide oxidoreductase family protein [Polyangia bacterium]	27.4	588	87.50%
	Select seq tpg|HAU80986.1| TPA: coagulation factor 5/8 type domain-containing protein [Stenotrophomonas sp.]	26.1	1673	100.00%
	Select seq gb|MBU6998043.1| penicillin acylase family protein [Theionarchaea archaeon]	26.1	1680	100.00%
ANDLLGPMWKHN	Select seq ref|WP_101773216.1| TIGR01212 family radical SAM protein [Peptostreptococcus faecalis]	29.5	238	87.50%
	Select seq gb|MDR3640519.1| adenylyl-sulfate reductase subunit alpha [Humidesulfovibrio sp.]	29.1	336	80.00%
	Select seq ref|WP_132878375.1| LLM class F420-dependent oxidoreductase [Tamaricihabitans halophyticus]	29.1	337	87.50%
	Select seq gb|ULT39904.1| CGNR zinc finger domain-containing protein [Niabella sp. I65]	29.1	338	80.00%
KLNKAGPPPLK	Select seq gb|KOX71108.1| Serine protease nudel [Melipona quadrifasciata]	28.6	475	100.00%
	Select seq emb|CAB62909.2| 1,5-bisphosphate carboxylase/oxygenase large subunit [Opilia sp. Chase 1902]	27.8	788	90.00%
	Select seq gb|AAC08698.1| ribulose-1,5-bisphosphate carboxylase/oxygenase large subunit [Opilia amentacea]	27.8	788	90.00%
	Select seq ref|WP_037678795.1| hypothetical protein [Streptomyces catenulae]	27.8	795	100.00%
SPHKKKKLPGAGG	Select seq ref|XP_013199631.2| forkhead box protein J1-B [Amyelois transitella]	31.2	71	100.00%
	Select seq ref|YP_010048434.1| ribosomal protein S4 [Lonicera fulvotomentosa]	31.2	72	100.00%
	Select seq ref|XP_053705038.1| protein kinase C-binding protein 1-like isoform X3 [Synchiropus splendidus]	29.9	202	90.91%
	Select seq gb|KAF8952262.1| peroxidase TAP [Flammula alnicola]	27.8	1160	100.00%
QPPALNDSYLYGPQ	Select seq gb|KAF8804039.1| Dyp-type peroxidase [Phlegmacium glaucopus]	27.8	1161	100.00%
	Select seq gb|MCY4450844.1| CmcJ/NvfI family oxidoreductase [Immundisolibacterales bacterium]	29.9	244	88.89%
QGPTAMKGPPYG	Select seq ref|XP_026223316.1| zinc finger CCCH domain-containing protein 4 isoform X1 [Anabas testudineus]	31.2	59	90.00%
	Select seq ref|XP_026223324.1| zinc finger CCCH domain-containing protein 4 isoform X2 [Anabas testudineus]	31.2	59	90.00%
	Select seq ref|XP_026223333.1| zinc finger CCCH domain-containing protein 4 isoform X3 [Anabas testudineus]	31.2	59	90.00%
